# The acid-sensing ion channel, ASIC2, promotes invasion and metastasis of colorectal cancer under acidosis by activating the calcineurin/NFAT1 axis

**DOI:** 10.1186/s13046-017-0599-9

**Published:** 2017-09-19

**Authors:** Zhi-hang Zhou, Jin-wen Song, Wen Li, Xue Liu, Liu Cao, Lu-ming Wan, Ying-xia Tan, Shou-ping Ji, Yu-mei Liang, Feng Gong

**Affiliations:** 1 Department of Pathology, the 309th hospital of PLA, Beijing, China; 2 Department of Tissue Engineering, Beijing Institute of Transfusion Medicine, Beijing, China; 3 Department of Pathology, Basic Science School, Jining Medical University, Jining, Shandong China; 4 Department of Surgery, the 15th hospital of PLA, Xinjiang, China

**Keywords:** Colorectal cancer, Acidosis, ASIC2, NFAT1, Metastasis

## Abstract

**Background:**

The tumor acidic microenvironment, a common biochemical event in solid tumors, offers evolutional advantage for tumors cells and even enhances their aggressive phenotype. However, little is known about the molecular mechanism underlying the acidic microenvironment-induced invasion and metastasis.

**Methods:**

We examined the expression of the acid-sending ion channel (ASIC) family members after acidic exposure using RT-PCR and immunofluoresence. Gene manipulation was applied to reveal the potential of ASIC2 on invasion, proliferation, colony formation of colorectal cancer (CRC). We assessed the in vivo tumor growth by subcutaneous transplantation and metastasis by spleen xenografts. Chromatin immunoprecipitation-sequencing was used to uncover the binding sites of NFAT1. Finally, we examined the expression of ASIC2 in CRC tissues using immunohistochemistry.

**Results:**

Acidic exposure led to up-regulation of the acid-sensing ion channel, ASIC2, in colorectal cancer (CRC) cells. ASIC2 overexpression in CRC cell lines, SW480 and HCT116, significantly enhanced cell proliferation in vitro and in vivo, while ASIC2 knockdown had the reverse effect. Importantly, ASIC2 promoted CRC cell invasion under acidosis in vitro and liver metastasis in vivo. Mechanistically, ASIC2 activated the calcineurin/NFAT1 signaling pathway under acidosis. Inhibition of the calcineurin/NFAT pathway by cyclosporine A (CsA) profoundly attenuated ASIC2-induced invasion under acidosis. ChIP-seq assay revealed that the nuclear factor, NFAT1, binds to genes clustered in pathways involved in Rho GTPase signaling and calcium signaling. Furthermore, immunohistochemistry showed that ASIC2 expression is increased in CRC samples compared to that in adjacent tissues, and ASIC2 expression correlates with T-stage, distant metastasis, recurrence, and poor prognosis.

**Conclusion:**

ASIC2 promotes metastasis of CRC cells by activating the calcineurin/NFAT1 pathway under acidosis and high expression of ASIC2 predicts poor outcomes of patients with CRC.

**Electronic supplementary material:**

The online version of this article (10.1186/s13046-017-0599-9) contains supplementary material, which is available to authorized users.

## Background

The acidic extracellular microenvironment, namely, acidosis due to the Warburg effect (aerobic glycolysis) and poor vasculature, is a biochemical hallmark of cancer [1]. The pH value of the extracellular fluid usually drops from 7.2–7.4 in the physiological state to 6.2–6.9 in solid tumors [2]. At present, acidosis contributes to the genetic instability of tumor cells and profoundly change their transcriptomic profiles, leading to phenotypes that are particularly suitable for survival, growth, and even metastasis in the acidic environment [3]. Although accumulating evidence demonstrated that acidosis affects cancer progression by promoting tumor cell migration, invasion, metastasis, and angiogenesis [4–7], the underlying mechanism is yet to be explored.

Acid-sensing ion channels (ASICs) are a proton-gated subgroup of the degenerin/epithelial sodium channel family of cation channels, and are widely expressed in the nervous system [8]. At least six ASIC subunit proteins (ASIC1a, ASIC1b, ASIC2, ASIC2b, ASIC3, and ASIC4), encoded by four genes, have been identified. Upon activation by protons, these channels play crucial roles in various physiological and pathological processes such as nociception, mechanosensation, learning and memory, and acidosis-mediated neuronal injury [9]. ASIC2 is less sensitive to proton in comparison to other ASIC family members. Recent studies indicated that ASICs are also expressed outside the nervous system (in dendritic cells, articular chondrocytes, and smooth muscle) and have important effects on physiological and pathological functions [9–12]. ASIC1 mediates acidosis-induced reactive oxidative species production and NF-κB signaling activation in breast cancer [13]. The authors also showed that ASIC1 is required for the growth and metastasis of breast cancer in vivo [13]. Moreover, ASIC2 knockdown aggravates acidosis-induced injury of rat C6 glioma cells, accompanied with increased intracellular Ca^2+^ [14], indicating the protective role of ASIC2 on glioma cells under acidosis by preventing calcium overload. However, the role of ASIC proteins in acidosis-induced growth and metastasis of colorectal cancer (CRC) remains elusive.

The calcineurin/nuclear factor of activated T cells (NFAT) is one of the main signals down-stream of calcium signaling. Although first identified as a transcription factor nearly three decades ago, the role of NFAT was not fully studied until the development of immunosuppressants such as cyclosporine (CsA) and tacrolimus (FK506). These two drugs block the activity of calcineurin, which is a phosphatase that dephosphorylate the hyper-phosphorylated NFAT factors and leads to their nuclear translocation [15]. Increasing studies showed that NFAT factors play essential role in tumor initiation and progression such as proliferation, invasion, migration, and angiogenesis [16]. Among the five members (NFAT1–5), NFAT1 is involved in cancer proliferation, invasion, apoptosis, angiogenesis, and lymphangiogenesis [15]. NFAT1 promoted invasion and migration of breast cancer cells, induced the transcription of oncogene murine double minute 2 (MDM2), and inactivated the tumor suppressor, p53 [17]. In addition, NFAT1 is overexpressed in lung cancer tissues and correlates with poor prognosis of patients with lung cancer [18]. Augmented expression of NFAT1 was also detected in glioblastoma multiforme and promoted invasion via MMP7/9 up-regulation [19].

In the present study, we investigated the effect of acidosis on the expression of ASIC family members in CRC cell lines and determined that ASIC2 was dramatically up-regulated by acidosis. Gain and loss of function studies indicated that ASIC2 promotes cell proliferation, invasion, and metastasis in CRC cell lines in vitro and in vivo*.* Further molecular studies, including ChIP-seq assay, indicated that ASIC2 activates the calcineurin/NFAT1 signaling pathway under acidosis and that NFAT1 binds to genes clustered in pathways involved in Rho GTPase signaling and calcium signaling. Furthermore, immunohistochemistry indicated that ASIC2 expression is increased in CRC samples and correlates with T-stage, distant metastasis, recurrence, and poor prognosis.

## Methods

### Cell culture and tissue samples

The human CRC cell lines, HCT116, SW480,LoVo, SW620, and HT29, were obtained from the American Type Culture Collection (ATCC, Manassas, VA, USA) and maintained in DMEM supplemented with 10% FBS (Gibco, Carlsbad, CA, USA) in a 5% CO_2_ atmosphere at 37 °C. The medium was further supplemented with HEPES and PIPES (25 mM each) and the pH was adjusted to 6.5.

CRC specimens were collected from 147 patients with CRC from 2008 to 2011 at the 309th hospital of PLA after informed consent was obtained from all patients. The patients did not receive chemotherapy or radiotherapy before surgery. The diagnoses of CRC were made independently by at least two histopathologists. This study was carried out according to the principles of the Helsinki Declaration and approved by the Ethical Committee of the 309th hospital of PLA.

### Expression vector and transfection

Lentivirus particles expressing short hairpin RNA (shRNA) against ASIC2 or control shRNA were produced by Sbo-Bio (Shanghai, China). Lentivirus particles expressing ASIC2 (Lv-ASIC2) or empty vector (Lv-vector) were also purchased from Sbo-Bio. Stably transfected cells expressing EGFP were isolated with a flow cytometric sorting system (BD FACS Aria II, Bedford, MA, USA).

### RNA extraction and qRT-PCR

Total RNA was extracted from cultured cells with TRIzol Reagent (Takara, Dalian, China). The extracted RNA was examined by UV spectrophotometry to determine the concentration and purity. The cDNA was obtained by incubating 0.5 μg RNA with PrimeScript RT Master Mix (Takara). PCR was performed in a CFX96 real-time PCR system (Bio-Rad, Hercules, CA, USA) using SYBR Green (Takara). The PCR primers used are listed in Additional file 1: Table S1.

### Western blotting

Total proteins were extracted in a lysis buffer (Beyotime, Shanghai, China) and the nuclear proteins were extracted using a nuclear protein extraction kit (Beyotime). The denatured proteins were separated by 10% SDS-PAGE and then transferred to a PVDF membrane (Millipore, Billerica, MA, USA) by using a wet transfer apparatus (Bio-Rad). The membrane was then blocked in 5% (*w*/*v*) skim milk in phosphate buffered saline containing 0.1% Tween 20 (PBS-T) for 2 h at room temperature. Subsequently, the membrane was incubated with antibodies against ASIC2 (Abcam, Cambridge, UK), NFAT1 (Cell Signaling, Beverly, MA, USA), β-actin (Sigma, St Louis, MO, USA), or Histone H3 (Abcam) overnight at 4 °C. The HRP-coupled anti-rabbit secondary antibody was used at a final dilution of 1:5000 visualized with an enhanced chemiluminescence (ECL) detection system (Thermo Scientific, Waltham, MA, USA).

### Trypan blue assays

Cells were plated at 3 × 10^5^ cells/mL in 12 well plates and treated with or without acidic medium for 24 h. Cell viability was measured by adding trypan blue (Beyotime) to cell sample solution onto a hemocytometer.

### Immunofluorescence staining

Cells were fixed with paraformaldehyde for 30 min, permeabilized with Triton X-100 for 10 min, and blocked with 2.5% bovine serum albumin for 1 h. Cells were then incubated with an anti-ASIC2 antibody for 1 h at room temperature, followed by a Cy3-conjugated secondary antibody for 1 h. The nucleus was stained with DAPI for 5 min. Images were captured with an inverted fluorescence microscope (PerkinElmer, Norwalk, CT, USA).

### Invasion assay

Cell invasion ability was determined in vitro using Matrigel-coated polycarbonate membranes (8 μm pore size) (BD Biosciences, Bedford, MA, USA). Briefly, 1 × 10^5^ CRC cells were suspended in 100 μL serum-free medium and seeded into the upper chamber, while, in the lower chamber, 600 μL fresh complete medium was added as the chemoattractant. For the treatment with CsA, the lower chamber was supplemented with 5 or 20 μM CsA. Cells were then incubated for 24 h and non-invading cells in the upper membrane were removed with a cotton swab. Cells on the lower membrane were fixed and stained with crystal violet (Beyotime) and counted by examining five randomly selected fields.

### Cell proliferation assay

Cells were seeded at 1000 cells/well in 96-well plates in a final volume of 100 μL of culture medium per well. Cell proliferation was examined on day 1, 2, 3, 4, and 5. At each time point, 100 μL fresh culture medium containing 10 μL CCK-8 (Beyotime) were used to replace the culture medium and incubated for 2 h at 37 °C in 5% CO_2_ atmosphere. The absorbance was measured at a wavelength of 450 nm with a microplate reader (Thermo Scientific). All assays were repeated three times.

### Colony formation assay

Two hundred viable cells per well were seeded in 6-well plates and cultured in DMEM containing 10% FBS. After incubation for two weeks, colonies containing more than 50 cells were counted after crystal violet staining.

### Animal experiments

All animal experiments were performed in accordance with Institutional Animal Care and Use Committee approved protocols. To evaluate the in vivo tumorigenic effects, 1 × 10^6^ cells were suspended in serum-free-DMEM/Matrigel mixture (1:1 volume) and injected subcutaneously into the back of BALB/c nude mice. After 3 weeks, mice were euthanized, and xenografts were harvested and weighed. For in vivo liver metastasis assay, 1 × 10^6^ cells suspended in 50 μL of phosphate-buffered saline were injected into the distal tip of the spleen. The animals were euthanized after 6 weeks, and the spleen and liver were dissected out and fixed with formalin for pathological examination. Sections (5-μm thickness) of the liver were made at 10 different layers to cover the entire organ and stained with hematoxylin and eosin (H&E). Metastatic nodules were counted under microscopy in a double-blinded manner.

### High-throughput ChIP sequencing (ChIP-seq)

ChIP assay was performed by using a ChIP assay kit (Cell Signaling). Briefly, SW620 cells were crosslinked with formaldehyde then chromatin was fragmented by digestion with Micrococcal Nuclease and sonication. Anti-NFAT1 or rabbit anti-IgG antibodies were used for precipitation, and the immunoprecipitates were purified with Agarose Beads. The purified ChIP DNA was adapter ligated and PCR amplified according to the manufacturer’s instructions (Illumina, San Diego, CA, USA). High-throughput sequencing was performed on an Illumina Genome Analyzer and short reads were mapped to the human reference genome. Peaks were assigned to specific genes by proximity to the nearest transcription start site. The differential peak-associated genes were annotated and clustered according to the KEGG pathway and Reactome databases.

### Immunohistochemical staining

Following deparaffinization and rehydration, tumor sections (3-μm thickness) were incubated in 0.3% H_2_O_2_ in methanol for 30 min at 37 °C to block endogenous peroxidase. The sections were then boiled in 10 mmol/L citrate buffer (pH 6.0) for 2 min in an autoclave. The anti-ASIC2 (Abcam) or anti-NFAT1 (Cell Signaling) antibody was added and the sections were incubated at 4 °C overnight. The sections were visualized by using the diaminobenzidine solution (DAKO, Carpinteria, CA, USA), and then lightly counterstained with hematoxylin. Sections without incubation with primary antibody served as negative controls. The intensity of staining (brown color) was semi-quantitatively scored as follows: 1, weak; 2, medium; 3, strong; and 4, very strong. The percentage of maximally stained tumor cells in each section was recorded (0, <5%; 1, 5–30%; 2, 30–50%; 3, >50%). High expression of ASIC2 was defined as a combined score for the intensity and area of staining that was larger than 4. The results were verified by two pathologists independently.

### Statistical analysis

All statistics were performed using SPSS18.0 software (SPSS, Chicago, IL, USA). Measurement data are represented as mean ± SEM. Difference between groups was determined for statistical significance using Mann-Whitney test or Student’s t-test. *P* values less than 0.05 were considered significant. Survival analysis was assessed by using the Kaplan-Meier method, and survival rate was compared by log rank test.

## Results

### Acute acidosis induces ASIC2 expression

**Fig. 1:**
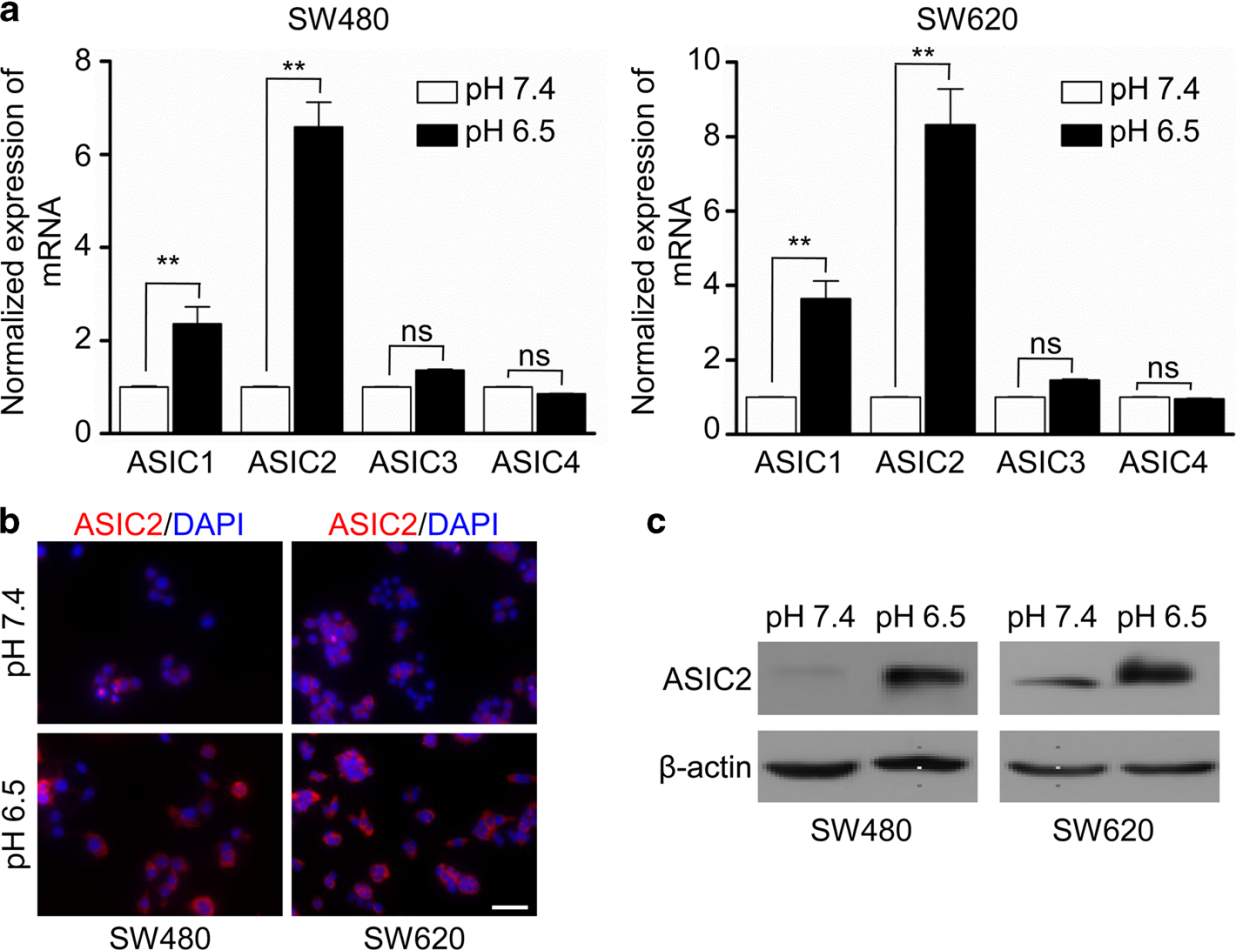
The acidic environment leads to increased expression of ASIC2. SW480 and SW620 cells were cultured in pH 6.5 medium for 24 h. **a** *ASIC1, ASIC2, ASIC3*, and *ASIC4* mRNA expression in SW480 or SW620 cells was analyzed by qRT-PCR. Data are shown as the fold change of expression in the acidic medium compared with that in normal medium (mean ± SEM). **b** Immunofluorescence staining was used to determined ASIC2 expression in SW480 and SW620 cells after acidic treatment. **c** Western blotting was performed to determine ASIC2 expression in SW480 and SW620 cells after acidic treatment. Results are representative of three independent experiments. **p < 0.01 compared with control. Scale bar, 200 μM

In this study, we first examined the effect of acidosis on the expression of ASICs. SW480 and SW620 cells were cultured in medium at pH 6.5 for 24 h. There was no significant change in cell viability detected by trypan blue (Additional file 2: Fig. S1A). RT-PCR results showed that acidosis significantly induced *ASIC2* expression (Fig. 1a). Notably, acidosis moderately induced the expression of *ASIC1*, but not that of *ASIC3* and *ASIC4.* Acidosis-induced ASIC2 expression was further validated by immunofluorescence and western blotting (Fig. 1b and c). Taken together, these results demonstrate that acidosis induces the expression of ASIC2 in CRC cells.

### ASIC2 promotes acidosis-induced CRC cell invasion

**Fig. 2:**
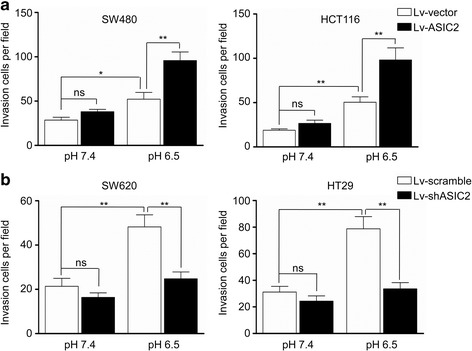
ASIC2 promotes invasion of CRC cells under acidosis. **a** SW480 and HCT116 cells expressing ASIC2 or vector were cultured in pH 6.5 medium for 24 h. The invasion ability was measured by Transwell invasion assay. **b** The invasion ability of SW620 and HT29 cells transfected with lentivirus expressing shASIC2 or scramble cultured in pH 6.5 medium for 24 h was measured by Transwell invasion assay. *p < 0.05, **p < 0.01

Previous studies showed that acidosis promotes the invasion of CRC cells [5]. To clarify the role of ASIC2 in acidosis-induced invasion, we first examined ASIC2 expression in five CRC cell lines by qRT-PCR and western blotting (Additional file 2: Fig. S1B and 1C). ASIC2 was highly expressed in HT29 and SW620 cells, while weakly expressed in SW480 and HCT116 cells. HT29 and SW620 cells were stably transfected with shASIC2 lentivirus vector, while SW480 and HCT116 cells were stably transfected with the ASIC2 expressing vector. Western blotting and qRT-PCR confirmed ASIC2 overexpression and knockdown in CRC cells (Additional file 3: Fig. S2). There was no significant change in cell viability after transfection (Additional file 4: Fig. S3A and 3B). Next, we measured the invasion capacity of CRC cells under acidosis. Although ASIC2 overexpression only slightly increased the invasion of CRC cells under normal pH conditions, it significantly potentiated the invasion of SW480 and HCT116 cells under acidosis (Fig. 2a). Consistently, ASIC2 knockdown attenuated acidosis-induced invasion of SW620 and HT29 cells, but it led to slight decrease under normal pH conditions (Fig. 2b). Collectively, these results demonstrate that ASIC2 promotes invasion of CRC cells under acidosis.

### ASIC2 promotes proliferation of CRC cells in vitro and in vivo

**Fig. 3:**
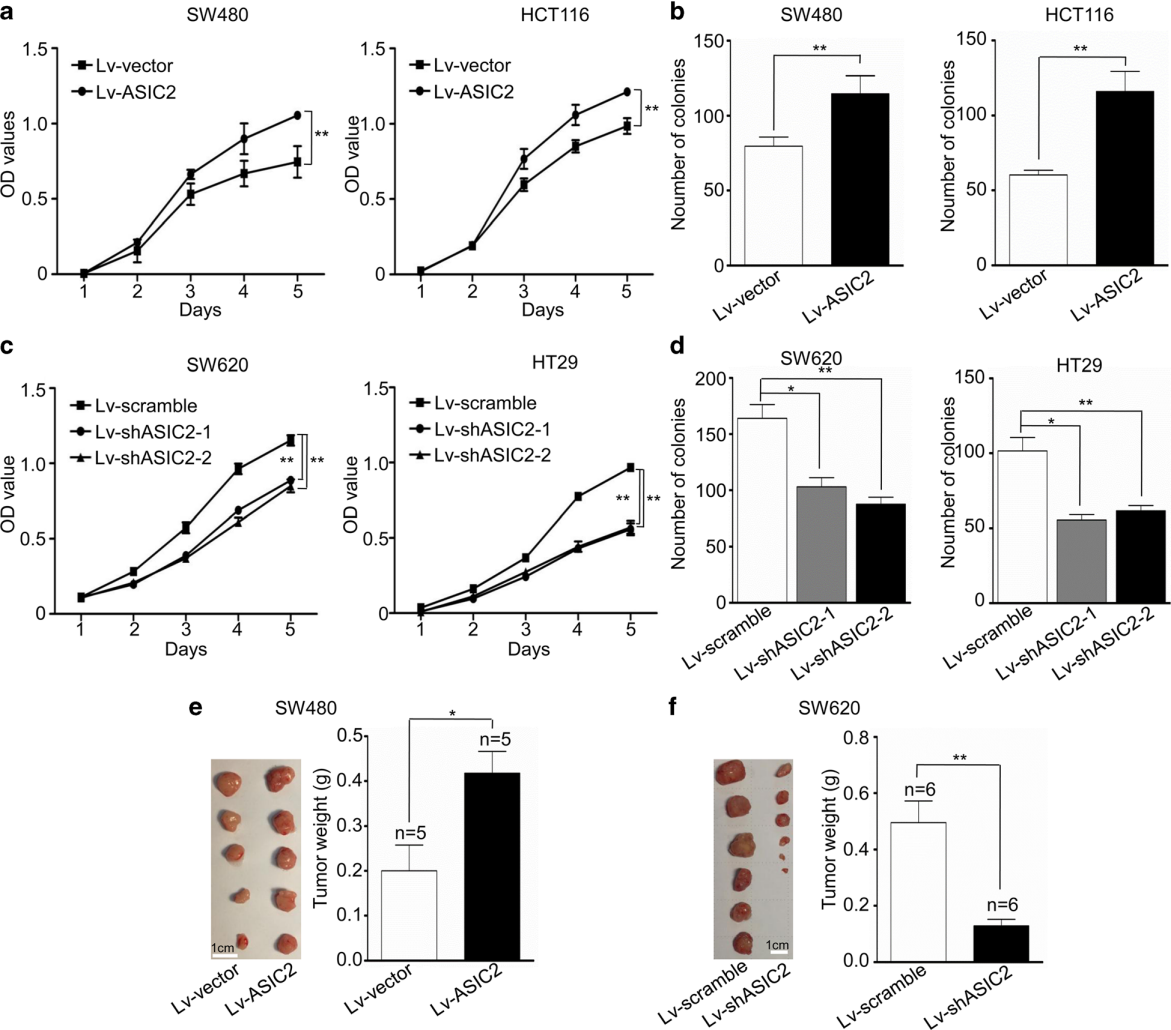
ASIC2 promotes proliferation and colony formation of CRC cells. **a** Proliferation of SW480 and HCT116 cells transfected with ASIC2 or vector were analyzed by CCK8 assay. The OD value was assessed at 1, 2, 3, 4, and 5 days, respectively. Each sample was tested in triplicates. **b** After 14 days of culture, the cells were stained with crystal violet and colonies containing more than 50 cells were counted. **c** Proliferation curve of SW620 (*left panel*) and HT29 (*right panel*) cells with or without ASIC2 knockdown. **d** Colony formation of SW620 (*left panel*) and HT29 (*right panel*) cells with or without ASIC2 knockdown. **e** SW480 cells transfected with ASIC2 or vector were injected subcutaneously into BALB/c nude mice. Mice were euthanized 3 weeks after transplantation, and tumors were excised and weighted. The tumors originated from SW480 cells transfected with ASIC2 were larger than that of control. Scale bar, 1 cm. **f** ASIC2 knockdown in SW620 cells inhibited tumor growth in vivo. Scale bar, 1 cm; *p < 0.05, **p < 0.01

CCK-8 and colony formation assays were used to determine the impact of ASIC2 on CRC cell proliferation in vitro. As shown in Fig. 3, ASIC2 overexpression promoted the proliferation (Fig. 3a) and colony formation (Fig. 3b, Additional file 4: Fig. S3C) of SW480 and HCT116 cells, while AISC2 knockdown inhibited the proliferation (Fig. 3c) and colony formation (Fig. 3d, Additional file 4: Fig. S3D) of SW620 and HT29 cells. To further determine whether ASIC2 could promote the growth of CRC cells in vivo, SW480 cells overexpressing ASIC2 were subcutaneously injected into BALB/c nude mice. Tumors originating from the Lv-ASIC2 group were larger than those originating from the Lv-vector group (Fig. 3e, *left panel*). The average weight of xenografts significantly increased from 0.20 g in the Lv-vector group to 0.42 g in the Lv-ASIC2 group (Fig. 3e, *right panel*). In contrast, SW620 cells expressing shASIC2 developed smaller tumors than the Lv-vector group (Fig. 3f, *left panel*). The average weight of xenografts was dramatically reduced from 0.50 g in the Lv-scramble group to 0.13 g in the Lv-shASIC2 group (Fig. 3f, *right panel*). These results indicate that ASIC2 promotes the proliferation of CRC cells in vitro and in vivo.

### ASIC2 promotes liver metastasis of CRC cells in vivo

**Fig. 4:**
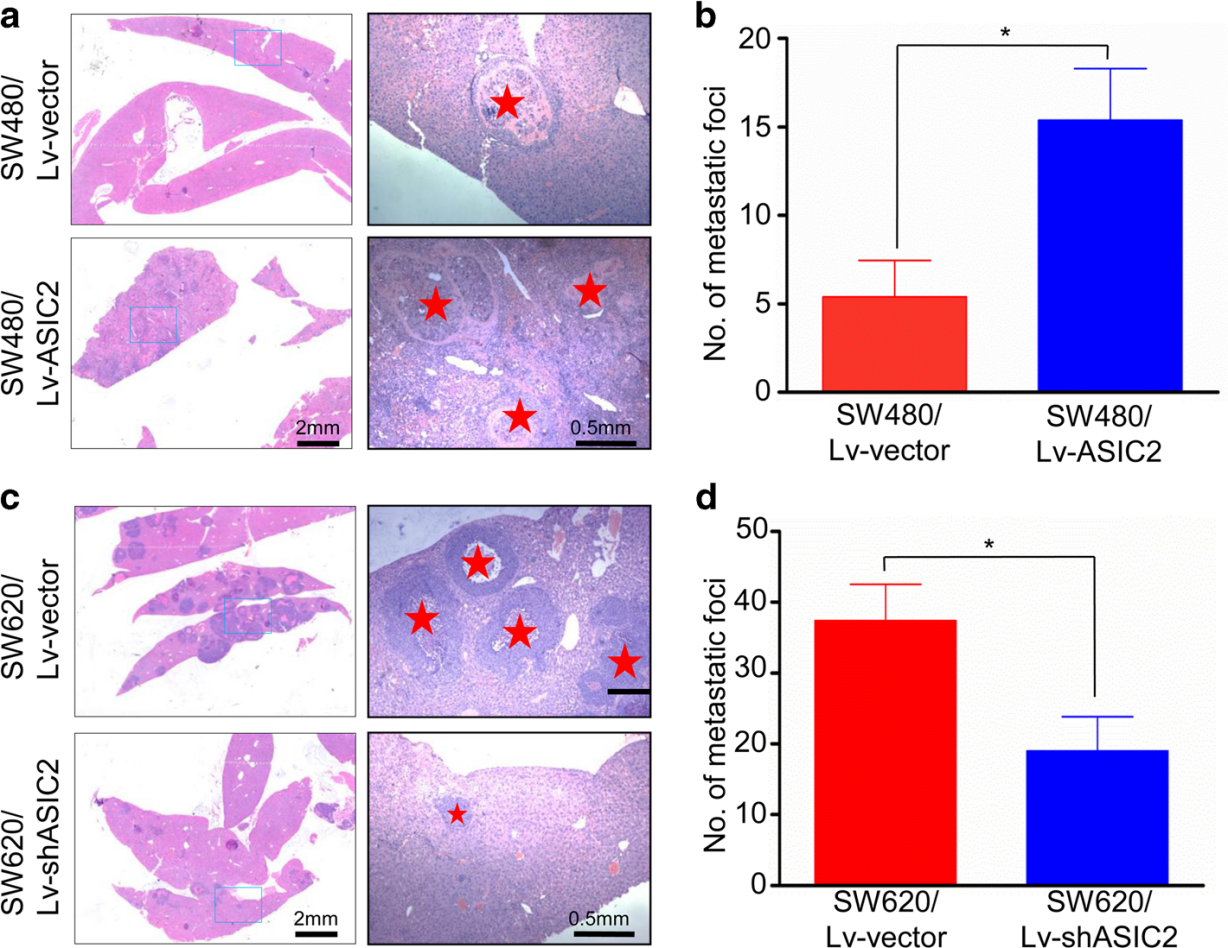
ASIC2 promotes metastasis of CRC cells in vivo. **a, b** Cells were injected into the distal tip of the spleen and mice were euthanized after 6 weeks. Metastatic nodules in the liver were counted. ASIC2 overexpression in SW480 cells resulted in more metastatic foci in the liver. **c, d** ASIC2 knockdown inhibited liver metastasis of SW620 cells. *p < 0.05, **p < 0.01

As ASIC2 is an H^+^-gated cation channel that might be activated by extracellular protons, and tumors usually present an acid extracellular pH, we hypothesized that the acidic extracellular microenvironment generated by CRC cells might promote metastasis by activating ASIC2 in vivo. CRC cells were injected into the spleen of BALB/c nude mice and the metastatic potential was measured by counting metastatic foci in the liver. Our results showed that ASIC2 overexpression promoted liver metastasis of SW480 cells (Fig. 4a), and significantly increased the average number of metastatic foci from 5.4 to 15.4 per liver (Fig. 4b). On the other hand, ASIC2 knockdown in SW620 cells reduced the average number of metastatic foci from 37.4 to 19 per liver (Fig. 4c and d). These results revealed that ASIC2 promotes liver metastasis of CRC cells in vivo*.*

### ASIC2 activates the calcineurin/NFAT1 axis under acidosis

**Fig. 5:**
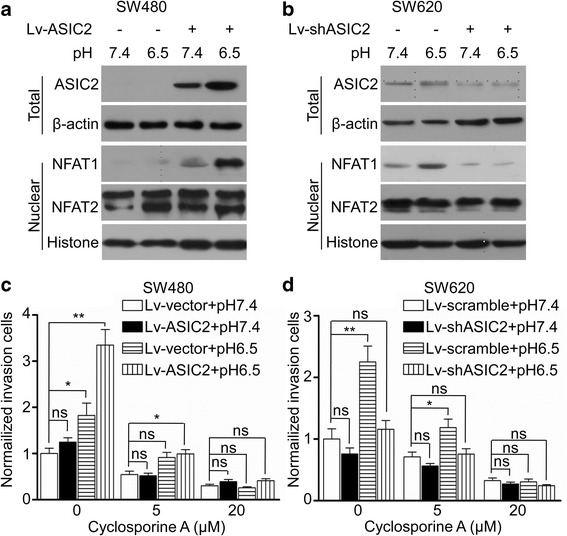
ASIC2 activates the calcineurin/NFAT1 pathway under acidosis. Cells were cultured at pH 7.4 and pH 6.5 for 24 h. The whole-cell extracts and nuclear extracts from **(a)** SW480 and **(b)** SW620 cells were used to analyze ASIC2 or NFAT1 by western blotting. Cell invasion ability was measured in the presence or absence of Cyclosporine A at the indicated concentration. **c** Cyclosporine A significantly inhibited ASIC2-induced invasion of SW480 cells under an acidic environment. **d** Cyclosporine A significantly inhibited acid-induced invasion of SW620 cells. *p < 0.05, **p < 0.01

Upon ASIC2 activation, calcium is elevated in the cytoplasm [20]. To explore the molecular mechanism of ASIC2-induced invasion and metastasis of CRC cells, we focused on the calcineurin/NFAT signaling pathway, which is one of the main downstream pathways of calcium signaling [21]. Our result showed that nuclear translocation of NFAT1 was slightly increased after acidic treatment for 24 h in SW480 and SW620 cells (Fig. 5a and b). ASIC2 overexpression remarkably increased the acidosis-induced nuclear translocation of NFAT1 (Fig. 5a), while ASIC2 knockdown had the opposite effect (Fig. 5b). It is worth noting that neither knock-down nor overexpression of ASIC2 influenced the expression of total NFAT1 (Additional file 4: Fig. S3E and 3F). ASIC2 manipulation did not affect the nuclear translocation of NFAT2 (Fig. 5a and b). Furthermore, inhibition of calcineurin/NFAT signaling by CsA significantly attenuated ASIC2-induced invasion of SW480 cells under acidosis in a dose-dependent manner (Fig. 5c). CsA also inhibited acidosis-induced invasion ability of SW620 cells (Fig. 5d). These results demonstrated that ASIC2 promotes invasion of CRC cells at least partially by activating the calcineurin/NFAT1 signaling pathway under acidosis.

### ChIP-seq analysis of the NFAT1 binding sites

**Fig. 6:**
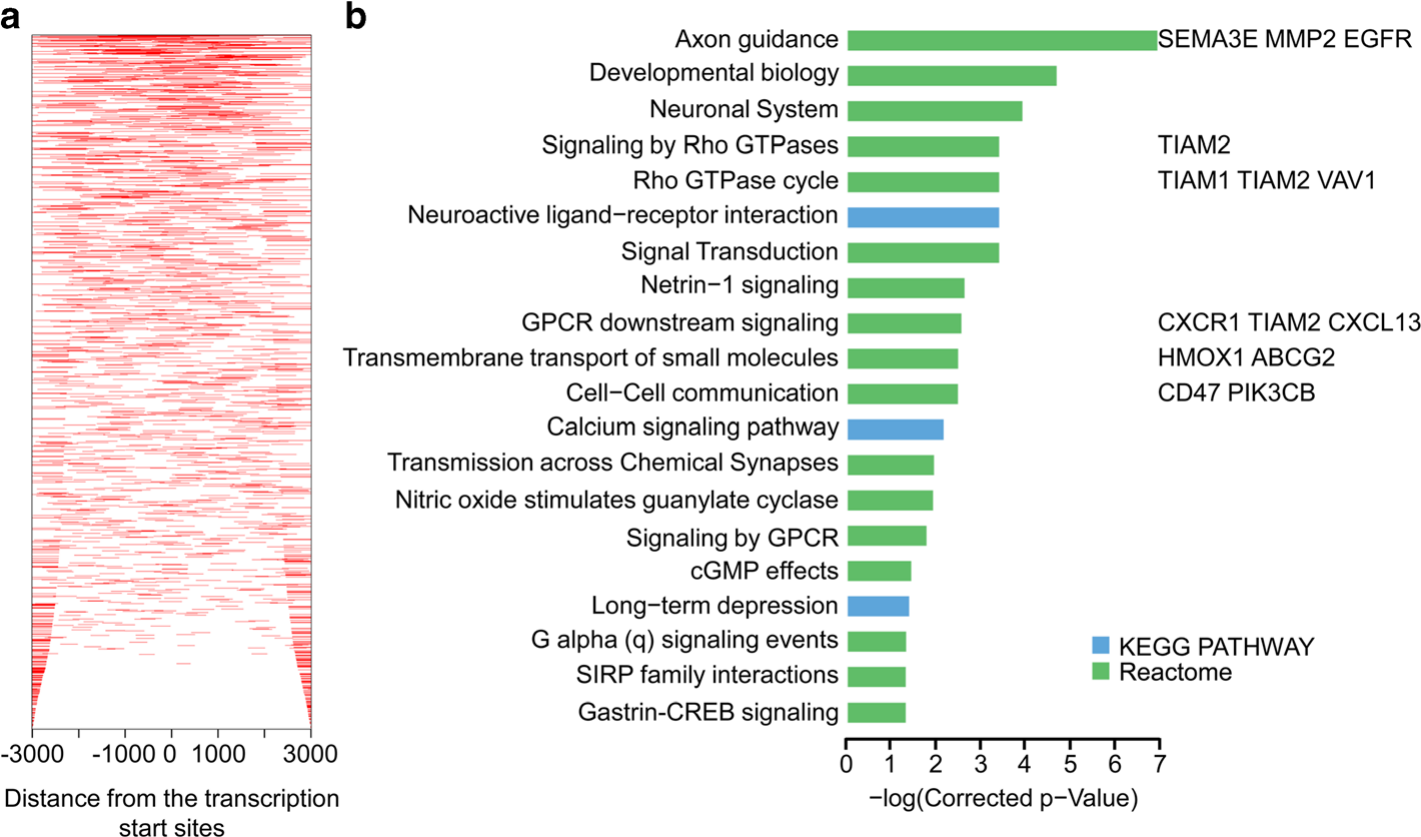
ChIP-Seq analysis of the NFAT1 binding sites. **a** ChIP-Seq density heatmap of NFAT1 for all RefSeq genes. The genomic region from -3 kb to +3 kb relative to the TSS is shown. **b** The top 20 enriched pathways of peak-related genes based on the KEGG or Reactome database

We utilized ChIP-seq assay to analyze the binding sites of NFAT1 in SW620 cells. High-throughput sequencing was performed on an Illumina Genome Analyzer and short reads were mapped to the human reference genome. Peaks were assigned to specific genes by proximity to the nearest transcription start site (TSS). The differential peak-associated genes were annotated and clustered according to the KEGG pathway and Reactome databases. The results showed that the binding sites of NFAT1 mainly located near the TSS (Fig. 6a), and revealed NFAT1-binding genes, including *SEMA3E, MMP2, EGFR, CXCR1, TIAM2*, and *PIK3CB* (Fig. 6b). These genes were clustered in pathways such as axon guidance, Rho GTPase, G-protein coupled receptors, and calcium signaling by KEGG pathway analysis (Fig. 6b). Silencing of NFAT1 resulted in down-regulation of CXCR1 and CD47 (Additional file 5: Fig. S4A). We also detected the expression of these genes after knock-down or overexpression of ASIC2. The results showed that ASIC2 enhanced the expression of CXCR1 under acidic microenvironment (Additional file 5: Fig. S4B and 4C). In addition, NFAT1 silencing inhibited the invasion of CRC cells (Additional file 6: Fig. S5A). These results suggest that NFAT1 plays an important role in the invasion, migration, and metastasis of CRC by regulating gene transcription.

### Increase of ASIC2 is correlated with poor clinical outcome in CRC patients

**Fig. 7:**
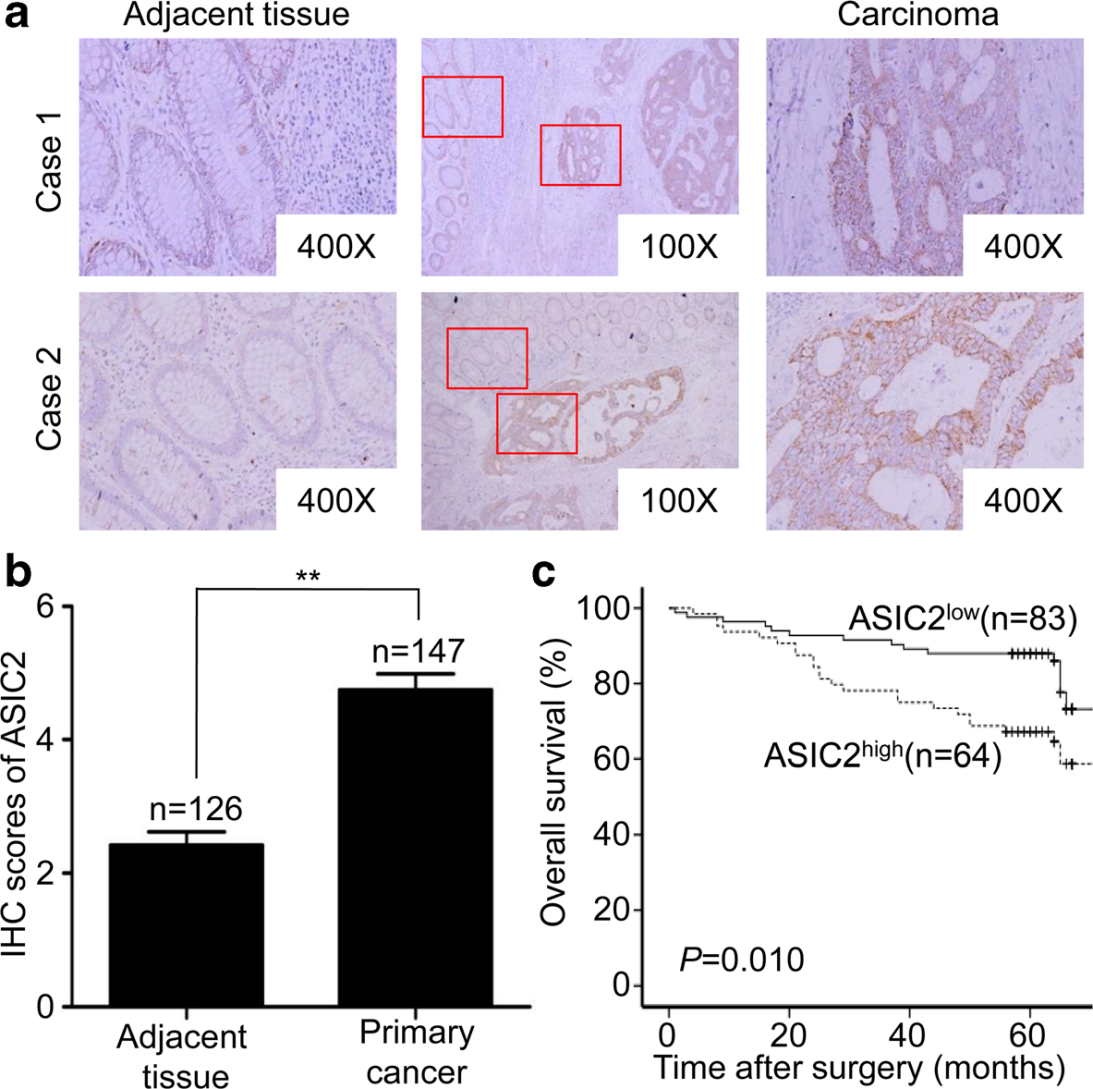
High expression of ASIC2 predicts poor outcomes of CRC patients. **a** Immunohistochemistry analysis was performed on paraffin-embedded tissues from 147 patients with CRC using a primary antibody against ASIC2. Representative images of ASIC2 staining in adjacent tissue (left panel) and carcinoma (right panel) are shown in CRC tissues (middle panel). **b** IHC scores of ASIC2 in primary cancer and adjacent tissues. ASIC2 expression was significantly increased in primary cancer. **c** Overall survival rates of CRC patients with high or low expression of ASIC2 were estimated by Kaplan-Meier analysis

Given the above results, we further analyzed the expression of ASIC2 and NFAT1 in 147 primary CRC tissues using immunohistochemistry. The clinical and pathological features of these patients are summarized in Table 1. ASIC2 was mainly expressed in the cell membrane and cytoplasm (Fig. 7a), while NFAT1 was expressed in the nucleus and cytoplasm (Additional file 6: Fig. S5B). High expression of ASIC2 was detected in 43.5% (64/147) of the CRC tissues. Positive nuclear NFAT1 was detected in 41.5% (61/147). Chi-square test showed that ASIC2 expression was associated with recurrence status, tumor stage, distant metastasis and NFAT1 positivity (Table 1). As shown in Fig. 7a, stronger staining of ASIC2 was observed in cancer cells than in adjacent normal tissue. The immunohistochemistry scores of ASIC2 were significantly higher than those in adjacent tissue (*p* < 0.01) (Fig. 7b). In addition, Kaplan-Meier analysis showed that high ASIC2 expression was associated with a shorter overall survival time (*p* = 0.010) (Fig. 7c). Taken together, these findings suggested that ASIC2 is associated with poor prognosis in patients with CRC.

**Table 1:** Correlation between ASIC2 expression and clinicopathological characters in colorectal cancer tissues from 147 patients

Clinicopathologic features		No. of patients (%)	ASIC2a status		*P* value
			Low (*n* = 83) No. (%)	High (*n* = 64) No. (%)	
Gender	Male	90 (61.2)	44 (48.9)	46 (51.1)	**0.020**
	Female	57 (38.8)	39 (68.4)	18 (31.6)	
Age	≤58	66 (44.9)	44 (66.7)	22 (33.3)	**0.024**
	>58	81 (55.1)	39 (48.1)	42 (51.9)	
Location	Rectum	78 (53.0)	46 (59.0)	32 (41.0)	0.514
	Colon	69 (46.9)	37 (53.6)	32 (46.4)	
Histological classification	Tubular	131 (89.1)	73 (55.7)	58 (44.3)	0.606
	Mucinous	16 (10.9)	10 (62.5)	6 (37.5)	
Tumor size (cm)	≤4	74 (50.0)	40 (54.1)	34 (45.9)	0.553
	>4	73 (50.0)	43 (58.9)	30 (41.1)	
Differentiation degree	Well	16 (10.9)	9 (56.3)	7 (43.7)	0.940
	Moderate	103 (70.1)	59 (57.3)	44 (42.7)	
	Poor	28 (19.0)	15 (53.6)	13 (46.4)	
Recurrence status	Positive	22 (15.0)	4 (18.2)	18 (81.8)	**0.000**
	Negative	125 (85.0)	79 (63.2)	46 (36.8)	
T-stage	T1	4 (2.7)	1 (25.0)	3 (75.0)	**0.040**
	T2	56 (38.1)	39 (69.6)	17 (30.4)	
	T3	83 (56.5)	42 (50.6)	41 (49.4)	
	T4	4 (2.7)	1 (25.0)	3 (75.0)	
Lymph node metastasis	Negative	117 (79.6)	67 (57.3)	50 (42.7)	0.698
	Positive	30 (20.4)	16 (53.3)	14 (46.7)	
Distant metastasis	M0	111 (75.5)	68 (61.3)	43 (38.7)	**0.039**
	M1	36 (29.4)	15 (41.7)	21 (58.3)	
TNM stage	I	47 (32.0)	32 (68.1)	15 (31.9)	0.084
	II	49 (33.3)	26 (53.1)	23 (46.9)	
	III	15 (10.2)	10 (66.7)	5 (33.3)	
	IV	36 (24.5)	15 (41.7)	21 (58.3)	
NFAT1	Negative	86 (58.5)	56 (65.1)	30 (34.9)	**0.018**
	Positive	61 (41.5)	27 (44.3)	34 (55.7)

## Discussion

Tumor heterogeneity poses one of the main challenges for cancer therapy [22]. The tumor microenvironment remains one of the drivers causing heterogeneity [23]. As tumor cells tend to utilize aerobic glycolysis and present poor vasculature, the extracellular fluid in tumor tissues is often acidic, an event known as acidosis [24]. The acidic tumor microenvironment functions as a selection pressure on all cells in the tumor tissues. The tumor cells surviving in the hostile microenvironment exhibit stronger invasion and metastasis potential [25]. In 2009, it was reported that reversing the acidic tumor microenvironment using bicarbonate inhibited metastasis of breast cancer cells in a mouse model [26]. Bicarbonate markedly enhanced the anticancer activity of transarterial chemoembolization for liver cancer in a clinical study [27]. The present study demonstrates that acidic stimulation leads to up-regulation of the acid sensing ion channel, ASIC2, which enhanced the invasion capacity of CRC cells.

It is speculated that ASIC2 does not mediate the response to extracellular acid within the physiological pH range as homomultimeric ASIC2 channels are much less pH-sensitive than other ASICSs (except ASIC4) [28]. ASIC2 expression was increased in neurons of the cortex and hippocampus, which were susceptible to ischemic injury, but not in neurons with detectable DNA damage [29]. This expression pattern was similar to that of anti-apoptotic proteins, BCL-2 and BCL-w, in surviving neurons, indicating that ASIC2 as a potential anti-apoptotic molecule. ASIC2 knockdown aggravates acidosis-induced injury of rat C6 glioma cells, accompanied with increased intracellular Ca^2+^ [14]. However, the expression and role of ASIC2 in CRC remains unknown. The present study demonstrates for the first time that ASIC2 overexpression enhances the progressive phenotype of CRC cells such as invasion and proliferation in vitro and in vivo, while ASIC2 knockdown had the opposite effect. It is worth noting that the effect of ASIC2 was not significant under normal pH conditions, which might be due to the low pH-sensitivity of ASIC2 [28]. We also showed that ASIC2 could activate the calcineurin/NFAT1 signaling and inhibition of this pathway reversed the acidosis-induced invasion of CRC cells.

Calcineurin, a Ca^2+^-dependent serine/threonine phosphatase with central functions in immunity, promotes intestinal tumor development by regulating the function of cancer stem cells in mice [30]. When activated by the increase of intracellular Ca^2+^, calcineurin dephosphorylates the NFAT proteins, leading to nuclear translocation of NFAT proteins [30]. Transcription factors of the NFAT family are important for various cellular processes, including T-cell activation, cardiac valve development, and osmotic stress response. Once in the nucleus, NFAT proteins form a complex with cell-type specific cofactors to control the transcription of target genes. NFAT1 is constitutively expressed in T cells and functions as a critical player in T-cell activation [31]. NFAT1-deficient mice showed significantly reduced IL-6 expression, which drives the development and progression of CRC [32, 33]. Moreover, the calcineurin/NFAT1 pathway is activated in triple-negative breast cancer and is essential for migration and invasion of breast cancer cells [21]. NFAT1 could bind to the promoter region of ADAMTS1 gene, which encodes a protease required for growth and metastasis of breast cancer cells [21]. Using ChIP-seq analysis, we found that NFAT1 binds to genes that are involved in pathways such as axon guidance, signaling by Rho GTPase [34], GPCR downstream signaling, cell-cell communication, and calcium signaling, implying a versatile role of NFAT1 in CRC. Among the genes to which NFAT1 binds, CXCR1 was induced by acidosis and ASIC2. CXCR1, also known as interleukin-8 receptor A, has been demonstrated to play important role in the proliferation, invasion, angiogenesis and metastasis of cancer cells [35]. Interestingly, we previously reported that SEMA3F, an axon guidance molecule, inhibited the growth and metastasis of CRC [36]. Whether NFAT1 could regulate the transcription of SEMA3F needs to be further investigated. As the regulatory mechanism by which acidosis induced the expression of ASIC2 is at present largely unknown, future work should be done to demonstrate it.

Finally, we determined the association between ASIC2 expression and clinicopathological features in CRC samples from 147 patients. The results revealed that high expression of ASIC2 correlated with recurrence, invasion depth, and distant metastasis. CRC patients with low ASIC2 expression tended to live longer than those with high expression of ASIC2. It should be note that the present study is limited to the acute effect of acidosis on the ASICs expression. Our future work will focus on the chronic effect of acidosis on ASICs expression and the underlying mechanism.

## Conclusions

Our results demonstrated that acidosis up-regulated ASIC2, which promotes growth and metastasis of CRC at least partially by activating calcineurin/NFAT1 signaling. The present study provides new evidence that acidosis promotes the aggressive phenotype of CRC cells at least partially through ASIC2 (Fig. 8). High ASIC2 expression predicts poor outcomes of CRC patients. Targeting ASIC2 may represent a novel therapeutic strategy for CRC treatment.

## Additional files

Additional file 1: **Table S1.** Primers used in the present study (DOCX 17 kb)

Additional file 2: **Fig. S1.** The expression of ASIC2 in CRC cell lines. (A) Cell viability detected by trypan blue staining following acidic exposure. (B) The mRNA expression of ASIC2 in CRC cell lines. (C) Western blot analysis of ASIC2 in CRC cell lines. ns, not significant (TIFF 644 kb)

Additional file 3: **Fig. S2.** Overexpression and knockdown of ASIC2 in CRC cell lines. The expression of ASIC2 in SW480 (A) and HCT116 (C) cells infected with Lv-vector and Lv-ASIC2 was examined by qRT-PCR and Western blotting analysis. The expression of ASIC2 in SW620 (B) and HT29 (D) cells infected with Lv-control, Lv-shASIC2–1 and Lv-shASIC2–2 was examined by qRT-PCR and Western blotting analysis. *p < 0.05, **p < 0.01 (TIFF 1329 kb)

Additional file 4: **Fig. S3.** ASIC2 enhances the colony formation capacity of CRC cells. (A, B) Cell viability detected by trypan blue staining after lentivirus transfection. (C) Overexpression of ASIC2 leads to enhanced colony formation capacity of SW480 and HCT116 cells. (D) Knockdown of ASIC2 attentuated the colony formation capacity of SW620 and HT29 cells. (E) Total NFAT1 expression in SW480 cells overexpressing ASIC2 with or without acidic exposure. (F) Total NFAT1 expression in SW620 cells expressing shASIC2 with or without acidic exposure. ns, not significant (TIFF 2616 kb)

Additional file 5: **Fig. S4.** ASIC2 enhanced the expression of CXCR1 under acidosis. (A) RT-PCR analysis of the expression of genes after silencing of NFAT1. (B) RT-PCR analysis of the expression of genes in SW480 cells overexpressing ASIC2 with or without acidic exposure. (C) RT-PCR analysis of the expression of genes in SW620 cells expressing shASIC2 with or without acidic exposure. *p < 0.05, **p < 0.01 (TIFF 1078 kb)

Additional file 6: **Fig. S5.** Silencing NFAT1 inhibited cell invasion. (A) NFAT1 silencing inhibited cell invasion with or without acidic exposure. (B) Representative images showing the expression of NFAT1 in CRC tissues. *p < 0.05, **p < 0.01 (TIFF 5351 kb)

## Abbreviations

ASIC2: Acid-sending ion channel
CRC: Colorectal cancer
CsA: Cyclosporine A
NFAT: Nuclear factor of activated T cells
ShRNA: Short hairpin RNA
