# Supplementary material for: The acid-sensing ion channel, ASIC2, promotes invasion and metastasis of colorectal cancer under acidosis by activating the calcineurin/NFAT1 axis
Source: J Exp Clin Cancer Res. 2017 Sep 19;36:130. doi: 10.1186/s13046-017-0599-9 (PMC5606037; doi:10.1186/s13046-017-0599-9)
Supplement: Supplementary file 1 — Table S1. Primers used in the present study (DOCX 17 kb) [file 13046_2017_599_MOESM1_ESM.docx]

**Table S1. Primers used in the present study.**

| Primer name | Sequence | Product length |
| --- | --- | --- |
| ASIC1-F | ATGGAAAGTGCTACACGTTCAA | 192 |
| ASIC1-R | GTTCATCCTGACTATGGATCTGC |  |
| ASIC2-F | GTGAGACCCGCTACATTGTGG | 129 |
| ASIC2-R | CCTTTTCCGCCAACAGACCT |  |
| ASIC3-F | CCAGTCCCACCTTTGACATGG | 123 |
| ASIC3-R | TGAAGATCGTGGTGAAGTTCTCA |  |
| ASIC4-F | CACTGGCCCTACTCACCTC | 159 |
| ASIC4-R | GGAAGCGGTTGATATTGCAGAG |  |
| NFAT1-F | GAGCCGAATGCACATAAGGTC | 107 |
| NFAT1-R | CCAGAGAGACTAGCAAGGGG |  |
| SEMA3E-F | AGGCAGGGACCTTGTATATTCC | 76 |
| SEMA3E-R | TGTACTCGGCCAGTGTATCTC |  |
| EGFR-F | AGGCACGAGTAACAAGCTCAC | 177 |
| EGFR-R | ATGAGGACATAACCAGCCACC |  |
| TIAM2-F | GTTCTGGAGCGAGCTAAAACT | 126 |
| TIAM2-R | GTACTTGAGCACTCTCTGAACC |  |
| TIAM1-F | GATCCACAGGAACTCCGAAGT | 121 |
| TIAM1-R | GCTCCCGAAGTCTTCTAGGGT |  |
| VAV1-F | CAACCTGCGTGAGGTCAAC | 159 |
| VAV1-R | ACCTTGCCAAAATCCTGCACA |  |
| CXCR1-F | CTGACCCAGAAGCGTCACTTG | 139 |
| CXCR1-R | CCAGGACCTCATAGCAAACTG |  |
| CXCL13 | GCTTGAGGTGTAGATGTGTCC | 83 |
| CXCL13 | CCCACGGGGCAAGATTTGAA |  |
| HMOX1-F | AAGACTGCGTTCCTGCTCAAC | 247 |
| HMOX1-R | AAAGCCCTACAGCAACTGTCG |  |
| ABCG2-F | ACGAACGGATTAACAGGGTCA | 93 |
| ABCG2-R | CTCCAGACACACCACGGAT |  |
| CD47 | AGAAGGTGAAACGATCATCGAGC | 160 |
| CD47 | CTCATCCATACCACCGGATCT |  |
| PIK3CB | TATTTGGACTTTGCGACAAGACT | 190 |
| PIK3CB | TCGAACGTACTGGTCTGGATAG |  |
| β-actin-F | CCTGTACGCCAACACAGTGC | 211 |
| β-actin-R | ATACTCCTGCTTGCTGATCC |  |
