# Supplementary figures and images for: The acid-sensing ion channel, ASIC2, promotes invasion and metastasis of colorectal cancer under acidosis by activating the calcineurin/NFAT1 axis
Source: J Exp Clin Cancer Res. 2017 Sep 19;36:130. doi: 10.1186/s13046-017-0599-9 (PMC5606037; doi:10.1186/s13046-017-0599-9)

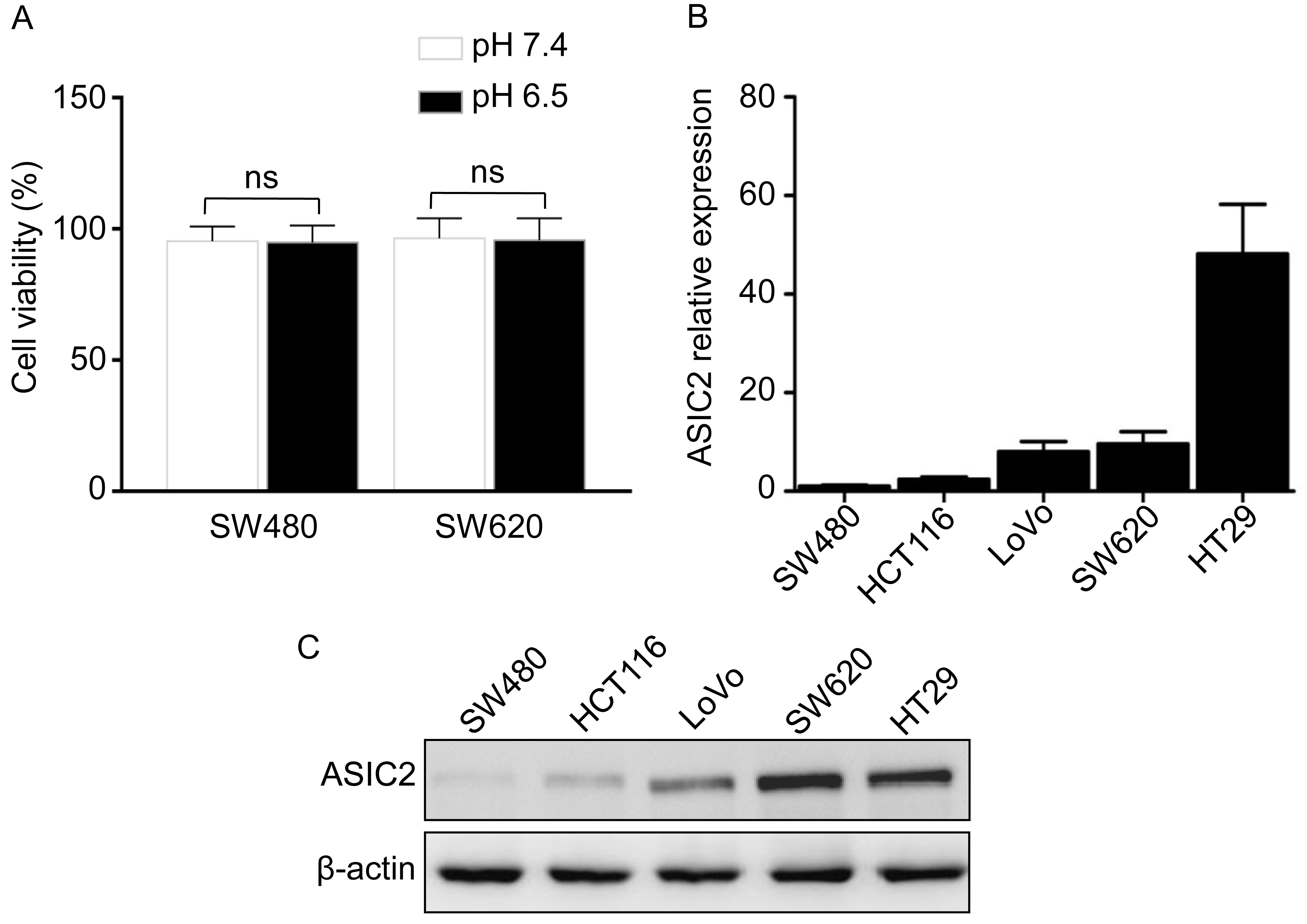

Supplement: Supplementary file 2 — Fig. S1. The expression of ASIC2 in CRC cell lines. (A) Cell viability detected by trypan blue staining following acidic exposure. (B) The mRNA expression of ASIC2 in CRC cell lines. (C) Western blot analysis of ASIC2 in CRC cell lines. ns, not significant (TIFF 644 kb) [file 13046_2017_599_MOESM2_ESM.tif]

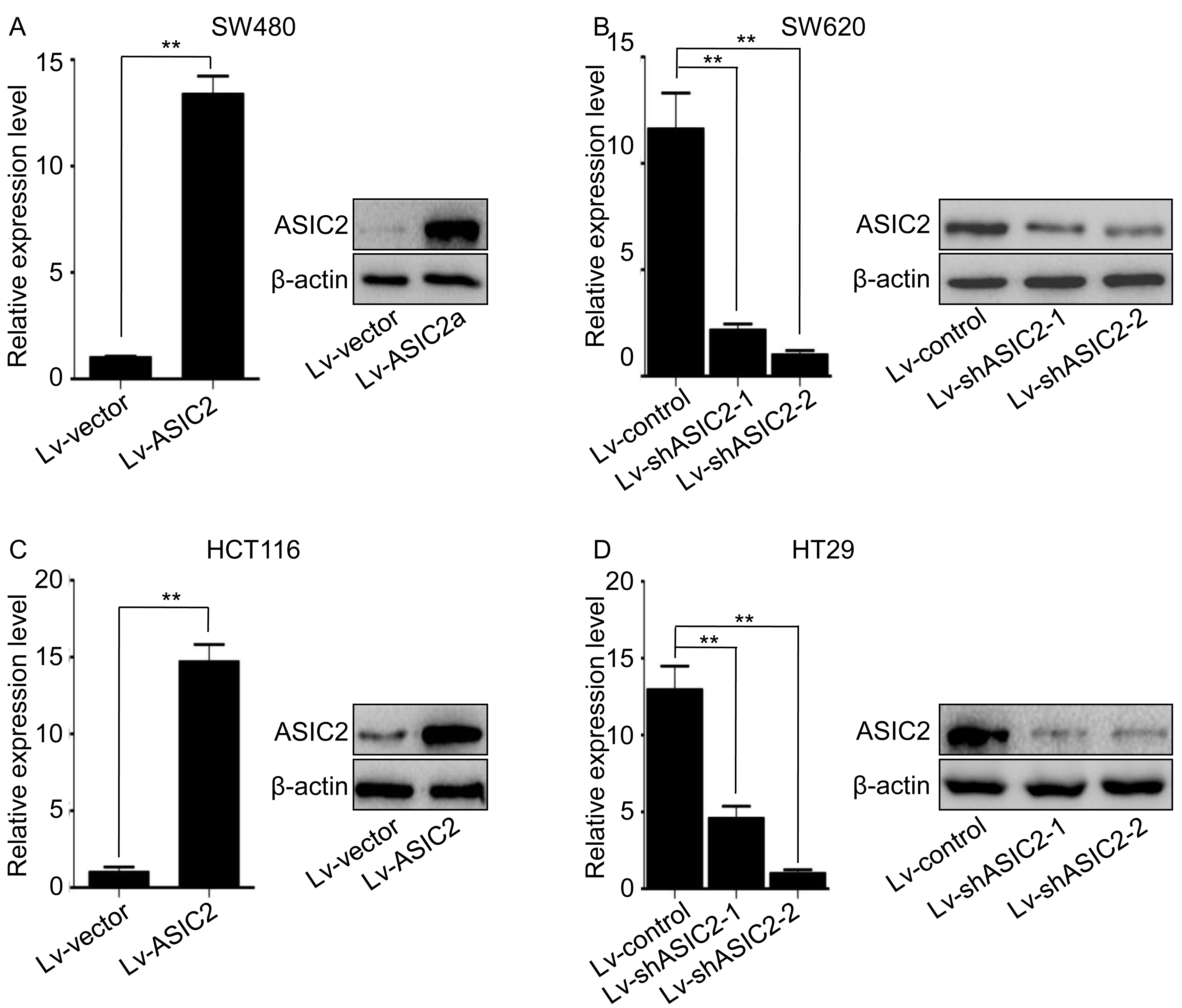

Supplement: Supplementary file 3 — Fig. S2. Overexpression and knockdown of ASIC2 in CRC cell lines. The expression of ASIC2 in SW480 (A) and HCT116 (C) cells infected with Lv-vector and Lv-ASIC2 was examined by qRT-PCR and Western blotting analysis. The expression of ASIC2 in SW620 (B) and HT29 (D) cells infected with Lv-control, Lv-shASIC2–1 and Lv-shASIC2–2 was examined by qRT-PCR and Western blotting analysis. *p < 0.05, **p < 0.01 (TIFF 1329 kb) [file 13046_2017_599_MOESM3_ESM.tif]

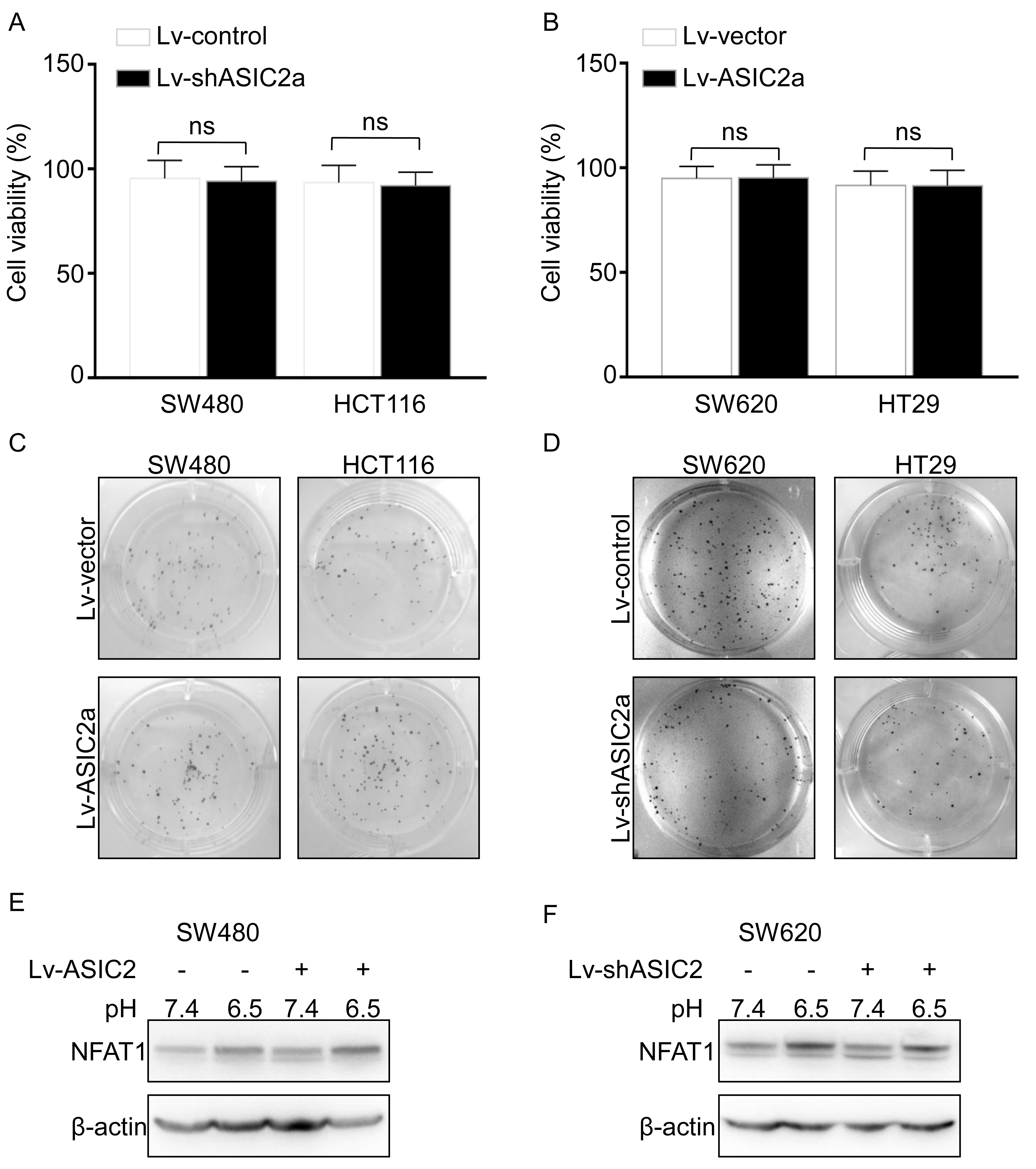

Supplement: Supplementary file 4 — Fig. S3. ASIC2 enhances the colony formation capacity of CRC cells. (A, B) Cell viability detected by trypan blue staining after lentivirus transfection. (C) Overexpression of ASIC2 leads to enhanced colony formation capacity of SW480 and HCT116 cells. (D) Knockdown of ASIC2 attentuated the colony formation capacity of SW620 and HT29 cells. (E) Total NFAT1 expression in SW480 cells overexpressing ASIC2 with or without acidic exposure. (F) Total NFAT1 expression in SW620 cells expressing shASIC2 with or without acidic exposure. ns, not significant (TIFF 2616 kb) [file 13046_2017_599_MOESM4_ESM.tif]

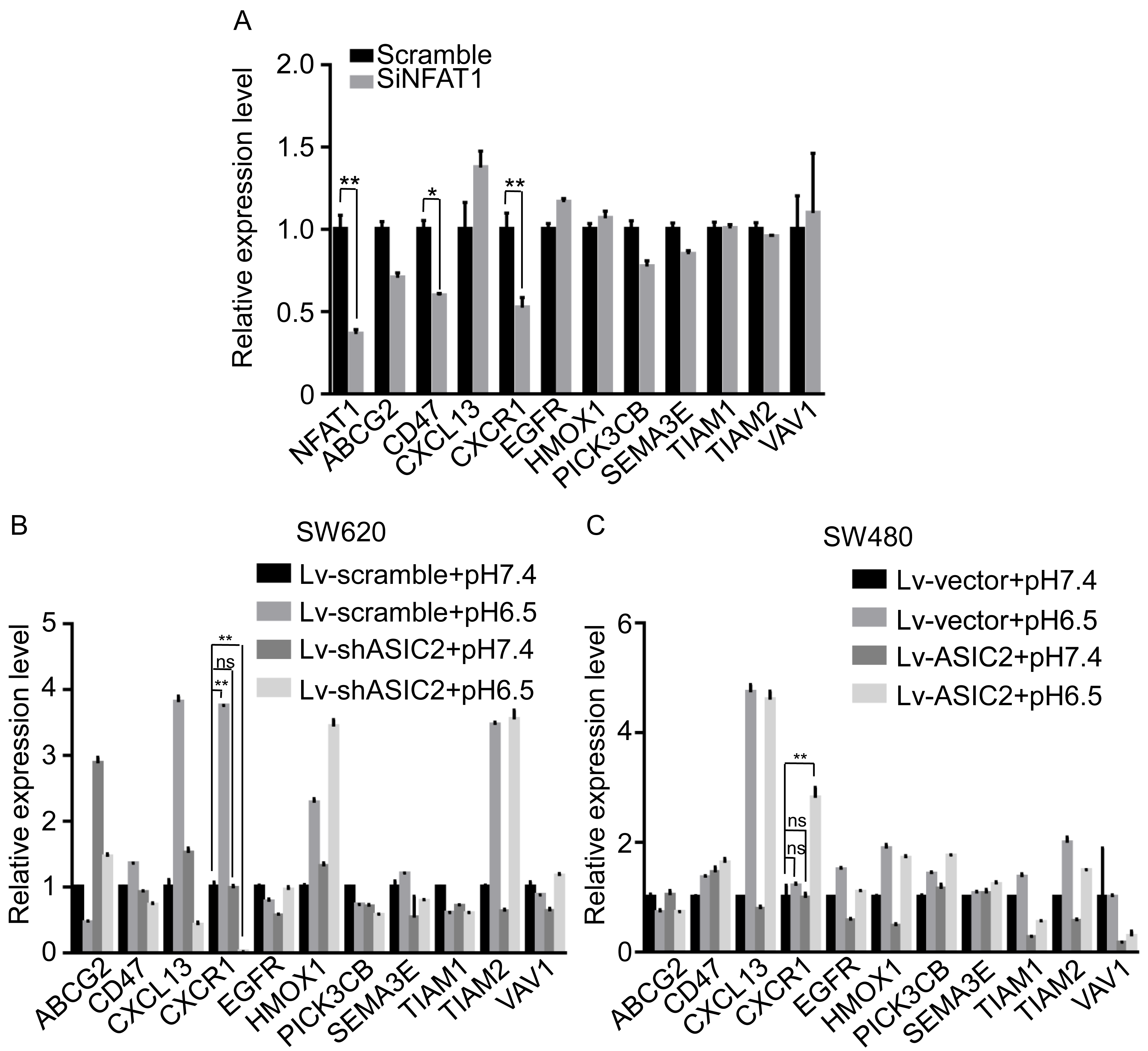

Supplement: Supplementary file 5 — Fig. S4. ASIC2 enhanced the expression of CXCR1 under acidosis. (A) RT-PCR analysis of the expression of genes after silencing of NFAT1. (B) RT-PCR analysis of the expression of genes in SW480 cells overexpressing ASIC2 with or without acidic exposure. (C) RT-PCR analysis of the expression of genes in SW620 cells expressing shASIC2 with or without acidic exposure. *p < 0.05, **p < 0.01 (TIFF 1078 kb) [file 13046_2017_599_MOESM5_ESM.tif]

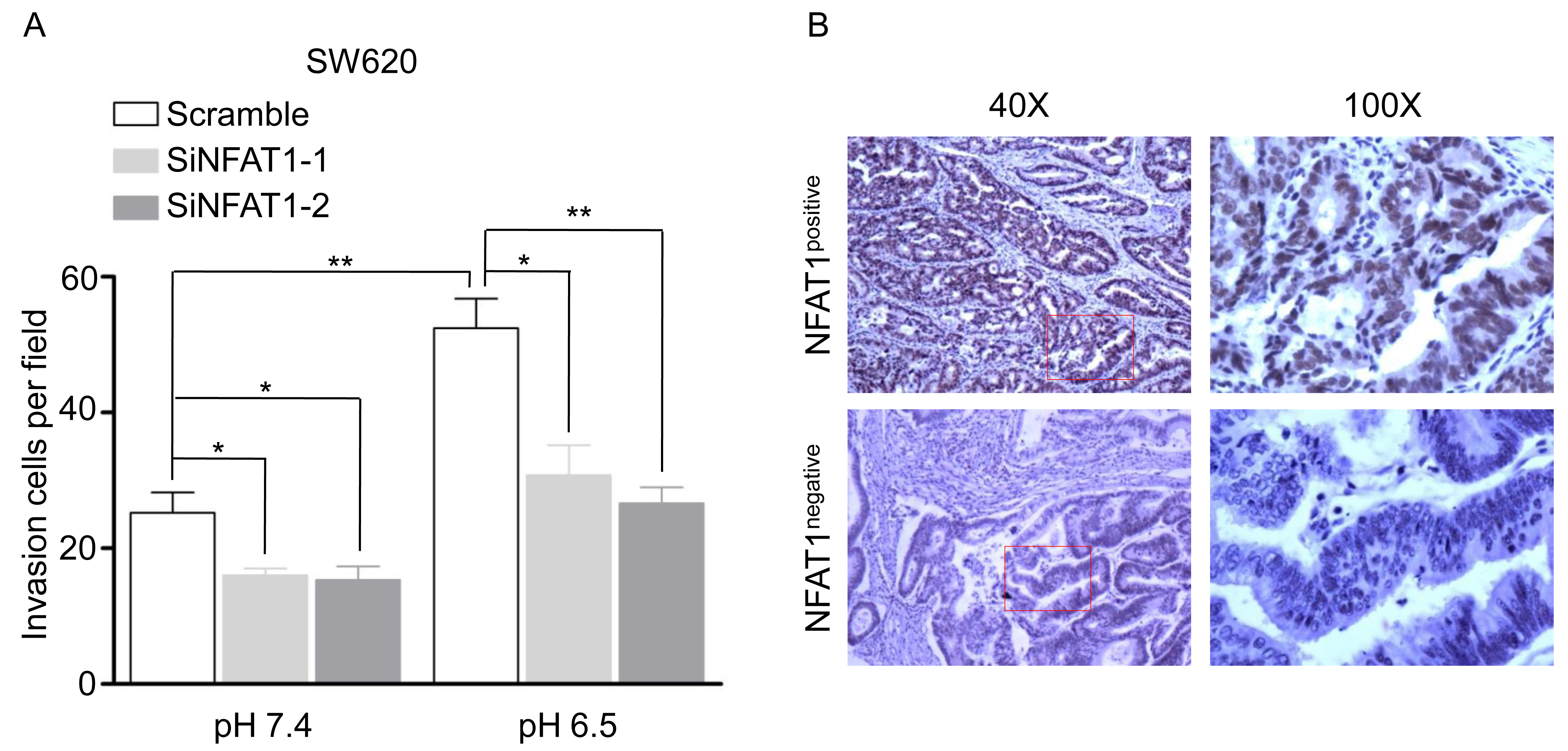

Supplement: Supplementary file 6 — Fig. S5. Silencing NFAT1 inhibited cell invasion. (A) NFAT1 silencing inhibited cell invasion with or without acidic exposure. (B) Representative images showing the expression of NFAT1 in CRC tissues. *p < 0.05, **p < 0.01 (TIFF 5351 kb) [file 13046_2017_599_MOESM6_ESM.tif]
